# Selectivity of the photosensitiser Tookad® for photodynamic therapy evaluated in the Syrian golden hamster cheek pouch tumour model

**DOI:** 10.1038/sj.bjc.6601428

**Published:** 2003-12-09

**Authors:** F Borle, A Radu, C Fontolliet, H van den Bergh, P Monnier, G Wagnières

**Affiliations:** 1 Institute of Environmental Engineering, Swiss Federal Institute of Technology (EPFL), CH-1015 Lausanne, Switzerland; 2 Department of Otolaryngology, Head and Neck Surgery, CHUV Hospital, CH-1011 Lausanne, Switzerland; 3 Institute of Pathology, University of Lausanne, CH-1011 Lausanne, Switzerland

**Keywords:** photosensitiser, photodynamic therapy, palladium-bacteriopheophorbide, Tookad®, pharmacodynamics, hamster tumour model, chemically induced squamous cell carcinoma

## Abstract

The response to photodynamic therapy (PDT) with the photosensitiser (PS) Tookad® was measured in the Syrian hamster cheek pouch model on normal mucosae and chemically induced squamous cell carcinoma. This PS is a palladium-bacteriopheophorbide presenting absorption peaks at 538 and 762 nm. The light dose, drug dose and drug injection-light irradiation times (DLI), ranging between 100 and 300 J cm^−2^, 1–5 mg kg^−1^ and 10–240 min respectively, were varied and the response to PDT was analysed by staging the macroscopic response and by the histological examination of the sections of the irradiated cheek pouch. A fast time decay of the tissular response with drug dose of 1–5 mg kg^−1^ was observed for DLI ranging from 10 to 240 min and for light doses of 100–300 J cm^−2^ delivered at a light dose rate of 150 mW cm^−2^. A significantly higher level of tissular response was observed for squamous cell carcinoma compared to normal tissue. Nevertheless, the threshold level of the drug–light dose for a detectable response was not significantly different in the tumoral *vs* normal tissue. The highest response at the shortest DLIs and the absence of measurable response at DLI larger than 240 min at light dose of 300 J cm^−2^ and drug dose of 5 mg kg^−1^ reveals the predominantly vascular effect of Tookad®. This observation suggests that Tookad® could be effective in PDT of vascularised lesions.

Photodynamic therapy (PDT) is developing as a treatment for cancer of the oesophagus (Radu *et al*, 2000), bronchi (Savary *et al*, 1997; Metz and Friedberg, 2001), bladder (Guillemin *et al*, 2001; Jichlinski and Leisinger, 2001) as well as for other nononcological applications such as the treatment of age-related macular degeneration with the recent approval of the benzoporphyrin derivative Visudyne® and Verteporfin® (Brown and Mellish, 2001). Photodynamic therapy is also a successful noninvasive therapeutic modality for treating cutaneous neoplasm (Fritsch *et al*, 1998; Karrer *et al*, 2001). Current photosensitisers (PS) (Photofrin®, Foscan® and Metvix®) accepted for the curative treatment of oncological indications still present strong drawbacks, such as prolonged skin (S) photosensitisation, scarring of healthy tissue, interpatient fluctuations, and intralesion heterogeneity (Grosjean *et al*, 1996; Webber *et al*, 1999). These drawbacks are due to the difficulties in predicting the response to the drug dose and to the lack of specificity for the targeted tissue Therefore, PDT is currently restricted to a limited number of clinical indications. The selectivity of a PS for neoplasm remains a main goal for new PSs. It has been reported that PSs of the pheophorbide type are accumulating in the reticuloendothelial system, the liver, the pancreas and the lungs, with a higher retention in pancreatic tumour cells (Aprahamian *et al*, 1993; Hajri *et al*, 1999). Photodynamic therapy of glioma xenografts with Pd-bacteriopheophorbide have been reported to decrease the development of metastasis in the rat (Schreiber *et al*, 2002). But frequently, no comparisons of the tissue response after PDT are reported between neoplasic *vs* healthy tissue under the same experimental conditions.

Tookad® is a palladium-metalated bacteriopheophorbide. It has an absorption spectrum typical for the bacteriochlorin macrocycle with a main absorption peak of the monomeric form in the near infrared at a wavelength of 762 nm. The presence of an absorption band at this wavelength allows treatment with a longer wavelength source, which results in a deeper penetration of the irradiation light (Ritz *et al*, 2001). This feature permits the more efficient treatment of solid tumours or pigmented tissues in particular if the interstitial approach is used (Chen *et al*, 2002). This advantage has been demonstrated by the deeper necrotic area obtained by a PS of the bacteriochlorin type (meta-(tetrahydroxy phenyl) bacteriochlorin (*m*THBPC), *λ*_absorption max_=740 nm) compared with the chlorin type (meta-(tetra-hydroxyphenyl) chlorin (*m*THPC), *λ*_absorption max_=652 nm) (Rovers *et al*, 2000). Finally, *in vitro* assays of bacteriochlorophyll derivatives demonstrate a higher phototoxic effect than haematoporphyrin derivative (Scherz *et al*, 2000; Rosenbach-Belkin *et al*, 1998).

These promising results with other bacteriochlorins triggered the present study on an *in vivo* animal model. The hamster cheek pouch model has been used for studies of the pharmacokinetics of PSs such as *m*THPC and benzoporphyrin derivative mono acid ring A (BPD-MA) (Andrejevic-Blant *et al*, 2000). The measurement of the phototoxic effect, of the pharmacodynamics, and the threshold of response on real physiological systems is a decisive factor for the development of future protocols for PDT and is essential in the early phase of development of new PSs. One problem inherent to PDT is the number of parameters that influence the therapeutic outcome. As it is difficult to optimise many parameters in a clinical context for each new PS, the use of an animal model provides relevant preclinical data for the clinical PDT trials. In order to optimise the therapeutic outcome of PDT, three parameters (drug dose (mg kg^−1^), drug injection–light irradiation time interval (DLI) (min) and the light dose (J cm^−2^)) were varied in the Syrian golden hamster healthy cheek pouch model and the resulting levels of response after treatment were measured. The selectivity of Tookad® against tumoral tissue was studied in respect to the response after simultaneous treatment on squamous cell carcinoma and on healthy mucosae in terms of drug dose, light dose and DLI. This model has previously been used with chemically induced squamous cell carcinomas and mimics well the carcinogenesis both macroscopically and microscopically of the human upper aerodigestive tract and oesophagus (Andrejevic-Blant *et al*, 1997a,^2001^; Kingsbury *et al*, 1997).

The PDT conditions for the study of selectivity on squamous cell carcinoma were then chosen so that the resulting levels of response on the healthy cheek pouch were limited to permit evaluation of the response of the tumour.

## MATERIALS AND METHODS

### Animals

A total of 144 Syrian golden hamsters (male, average weight 135±50 g) were used for the photodynamic treatments on the cheek pouch. Animals were housed at room temperature with a 12-h light/dark cycle. Food and drinking water were given *ad libitum*. The study of the light dose, drug–light intervals and drug dose was made using four animals per condition. In total, 120 animals were used for the normal tissue study and 24 animals for selectivity toward induced squamous cell carcinoma study. The animals used for the study of selectivity were submitted to chemical tumour induction on the left cheek pouch. The induction of squamous cell carcinoma was produced by a regular topical application (three times per week) on the mucosae of the left cheek pouch during 10 weeks with a 0.5% w v^−1^ oily 7,12−dimethylbenz(a)-anthracene (DMBA, Sigma Chemical, Buchs, Switzerland) following a previously described protocol (Andrejevic *et al*, 1996, Blant *et al*, 1996). At 12–14 weeks after the first application, the induced carcinoma were invasive, exophytic tumours with a diameter size of 2–8 mm. The right cheek pouch, which was not painted with DMBA, served for control purposes. All the experiments have been carried out with ethical committee approval and meet the standards required by the UKCCCR guidelines (Workman *et al*, 1998).

### Photodynamic treatments

#### Photosensitiser

The WST09, (CA Reg. No. 274679-00-4) produced under the name Tookad® by Steba-Biotech (2585 GB The Hague, The Netherlands), is a palladium-metalated bacteriopheophorbide. The solution of Tookad® was used directly as the stabilised micellar solution in aqueous medium provided by Negma-Lerads (Magny-Les-Hameaux, France) at 5 mg ml^−1^ (Batch No. L565331, Lot No. 010116). Solutions at 2 and 1 mg ml^−1^ were obtained by dilution in a formulation buffer at pH 7.4 (Negma-Lerads, Magny-Les-Hameaux, France).

The Tookad® solution was administered by intracardiac injection at 5, 2 or 1 mg kg^−1^ of body weight. Depending on animal weight, the injection volume was between 90 and 200 *μ*l. The precise intracardiac positioning of the needle was performed by carefully sucking some blood and monitoring the blood reflux in the syringe. All the injections and PDT experiments were performed under intraperitoneal anaesthesia (ketalar 150 mg kg^−1^ and xylesine 15 mg kg^−1^. The animals were killed 4 days after PDT treatment and the cheek pouch excised for macrophotography and histological preparation. All the experiments have been carried out with the ethical committee approval and meet the standards required by the UKCCCR guidelines (Workman *et al*, 1998).

#### Light source

Irradiation at 762 nm was performed with a tunable 4 W diode Laser Ceralas PDT 763 (CeramOptec GmbH, Bonn, Germany).

#### Light delivery

Photodynamic therapy on the mucosae of the hamster cheek pouch was carried out using a light distributor with a 12 mm diameter cylindrical radial diffuser, equipped with a circular side window of 1 cm^2^, producing a homogeneous irradiation onto the inner cheek pouch in direct contact with the diffuser as described in former publications (Blant *et al*, 1996; Andrejevic-Blant *et al*, 1997b). For the comparison of the normal and tumoral tissue, a simultaneous irradiation of both cheek pouches was achieved using a fibre splitter dividing the power in two equivalent part (CeramOptec GmbH, Bonn, Germany) and two light distributors. A light dose rate of 150 mW cm^−2^ was used for all treatments. The cylindrical diffusers were calibrated using an integration sphere by comparison with a frontal light diffuser (FD-1, Medlight SA, Ecublens, Switzerland) delivering 150 mW measured with a power-meter (Spectra Physics Power-meter 407A, Mountain View, CA, USA).

The light doses used for PDT were 100, 200 and 300 J cm^−2^ and DLIs of 10, 30, 120 and 240 min, defined as the duration between the end of injection and the beginning of the irradiation, were used.

### Histological preparation

The treated cheek pouches were resected, photographed, and then fixed in 5% buffered formalin (pH=7.0). The fixed specimens were paraffin embedded, sectioned in 5 *μ*m thick slices and stained with haematoxylin and eosin for histological examination. For each animal, the necrotic area was examined in serial sections to evaluate the depth of the histological necrosis extending to the different mucosal layers.

### Evaluation of the photodynamic effect

The assessment of the necrosis was made 4 days after PDT treatment. In order to evaluate the depth and extent of the PDT treatment, we used a tissue damage scale graded from 0 to 4. The scaling is based on the lateral extension of the macroscopic necrosis, the formation of fibrin and the in-depth extension of the necrosis to the different mucosal layers after histological examination of the tissues sections, in a similar way to the previous studies with different PSs (Andrejevic-Blant *et al*, 1997a,^1998^, Rosenbach-Belkin *et al*, 1998; Zellweger *et al*, 2000). Histologically, the tissue damage scale is related with the number of layers of tissue that are necrosed as well as the lateral extension of the necrosis. The grading of the response was expressed following the tissue damage scale summarised in Table 1

**Table 1:** Tissue damage scale

**Tissue damage scale**	**Macroscopically**	**Microscopically**
0	Absence of observable reaction	No tissue destruction
1	Erythema	Partial destruction of diffuse connective tissue (CT)
2	Necrosis with diameter< irradiation surface; no significant appearance of fibrin	Destruction of diffuse CT, lamina propria (LP); oedema of the tissue; beginning of the polymorphonuclear leucocyte invasion; partial loss of nuclei and striation in striated muscle (SM)
3	Necrosis and fibrin with diameter≈irradiation surface	Destruction of the mucosae epithelium (E), CT, and both muscular layer (SMI, SMO), strong polymorphonuclear invasion of these layers.
4	Necrosis and fibrin with diameter>irradiation surface	Destruction of all layers resulting in transmural necrosis

For the tissue damage scales 1 and 2, the epithelium was preserved. In these cases, the diameter of the necrosis was assessed through the epithelium due to its translucent properties.

.

### Statistical analysis

The contralateral irradiation of the normal cheek pouch allows a comparison of the level of response in each animal. The significance of the difference between tumour and normal tissue response was tested using the Wilcoxon matched-pairs signed-rank test. The tests were conducted using S-Plus software from Insightful Corporation (Seattle, WA, USA).

## RESULTS

### Macroscopic and histological aspects of the tissue reaction

The model of the Syrian hamster cheek pouch allows PDT treatment to be performed on a controlled surface with different layers of tissue, which are representative of the human aerodigestive tract. Following PDT treatment, an inflammatory reaction with oedema and necrosis developed during the following days. The analysis of the induced necrosis was made 4 days after PDT. The treatment of tumours requires a knowledge of the levels of response of the healthy tissue after the PDT in order to choose the conditions that are not highly necrotic for healthy tissue. Similar tissue damage scales were used in several studies of the PDT effects of other PSs such as Foscan® (Andrejevic-Blant *et al*, 1997a,^2000^).

In order to exclude all thermal effects due to the irradiation at 762 nm and any effect due to the formulation under irradiation, two blank series of four animals each were carried out: (a) irradiation at 762 nm with a light dose of 300 J cm^−2^ and a light dose rate of 150 mW cm^−2^ without PS and without injection of the formulation buffer; (b) irradiation at 762 nm with a light dose of 300 J cm^−2^ after injection of the formulation buffer without PS. In both blank series, no induced responses were observed (level 0), ensuring that all necrosis were effectively due to PDT treatment with Tookad®.

**Figure 1:**
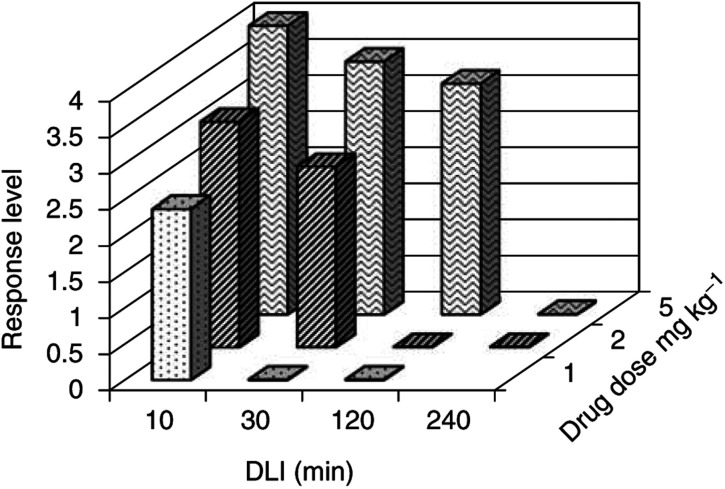
Response level of normal tissue after PDT at a light dose of 300 J cm^−2^.

The response level, as defined in Table 1, is presented in the form of a histogram as a function of the DLIs and drug doses (*D*) in Figures 1

, 2

**Figure 2:**
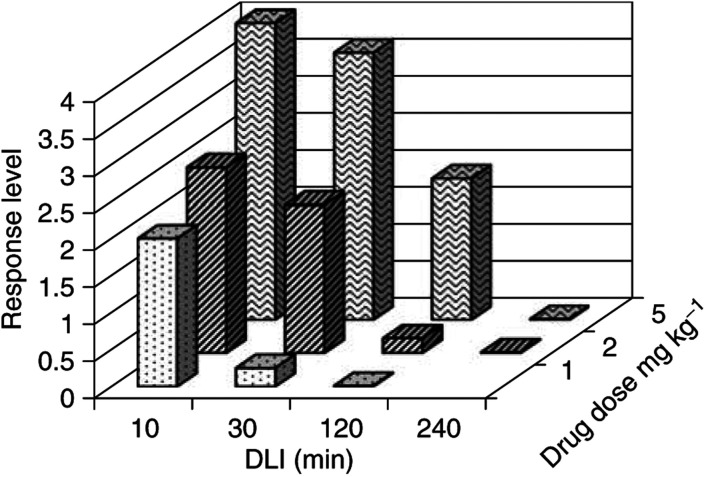
Response level of normal tissue after PDT at a light dose of 200 J cm^−2^.

and 3

**Figure 3:**
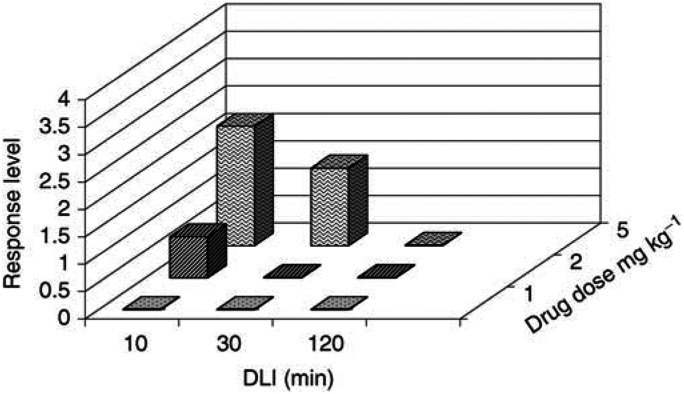
Response level of normal tissue after PDT at a light dose of 100 J cm^−2^.

, respectively, for light doses (*L*) of 300, 200 and 100 J cm^−2^. The typical standard deviation is ±0.6 on the tissue damage scale. The analysis of the response graphs (Figures 1, 2 and 3) show that, for each drug dose, a sharp decrease in response was observed for a certain DLI: for DLI>120 min at *D*=5 mg kg^−1^ and for DLI>30 min at *D*=2 mg kg^−1^. This corresponds to a threshold level of drug that is necessary to produce a measurable response after PDT. The response is limited to DLIs shorter than 240 min, indicating a fast clearance of Tookad® from the irradiated tissue and from the circulation.

**Figure 4:**
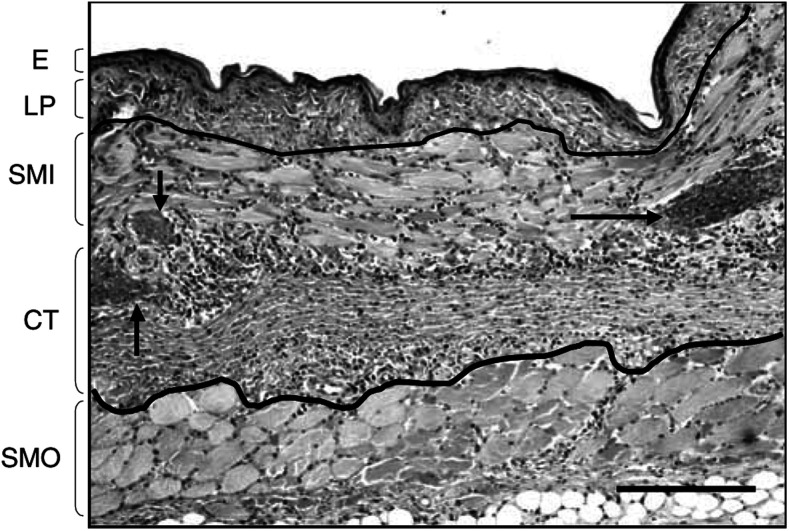
Histological section of the cheek pouch showing level 2 damage, with a necrosis of muscle and CT with polymorphonuclear invasion (between the black lines). An inflammatory oedema with stases of blood vessels indicated by arrows is present. The skin not visible in this figure, is located below the striated muscle layer. E=epithelium; LP=lamina propria; SMI=striated muscle inner layer; CT=connective tissue; SMO=striated muscle outer layer. The lower bar represents 200 *μ*m.

The different tissue layers are stacking in the mucosae in the following order from the irradiation side: epithelium (E), lamina propria (LP), inner striated muscle (SMI), diffuse connective tissue (CT), outer striated muscle (SMO), and skin (S). The histological analysis of the tissue sections has shown that the inflammatory response and necrosis appears unexpectedly at the level of the vascularised diffuse CT containing fibroblast, fibres of collagen and elastin between the SMI and SMO layer's and only at higher drug dose or higher light dose at the level of the inner epithelial layer (E). When necrosis is observed in the CT, it refers to the cellular content of this tissue, the inflammation being associated with an invasion by neutrophils. All necroses from response level 1 and 2 occur, therefore, in the submucosal layer first. The epithelial tissue of the mucosae, which is in direct contact with the light diffuser was preserved at the level of response 1 and 2 and presented no damage as shown in the histological section (Figure 4

). This observation can be explained by a lower vascularisation of the epithelium layer, than the CT layer, and therefore a lower local concentration of the PS at these short DLIs. At response level 2, the histological sections often present vascular occlusions and stases as well as the beginning of an invasion by the polymorphonuclear cells. At a response level 3, the necrosis reaches the epithelial tissue and all layers of muscle as well as the CT. At level 4, the necrosis extends across all the tissues, reaching the external epithelium (outside skin) corresponding to a transmural necrosis. At both levels 3 and 4, the extensive necrosis is associated with extensive cell death, disappearance of the muscle microstructure, and strong invasion by polymorphonuclear cells, as well as an oedema of all the tissue layers.

From these observations, a contrast of the necrosis for the vascularised diffuse CT and SM was induced at response level's 1 and 2 following PDT treatment.

### Selectivity of the phototherapeutic effect of Tookad® on chemically induced squamous cell carcinoma *vs* normal mucosae

**Figure 5:**
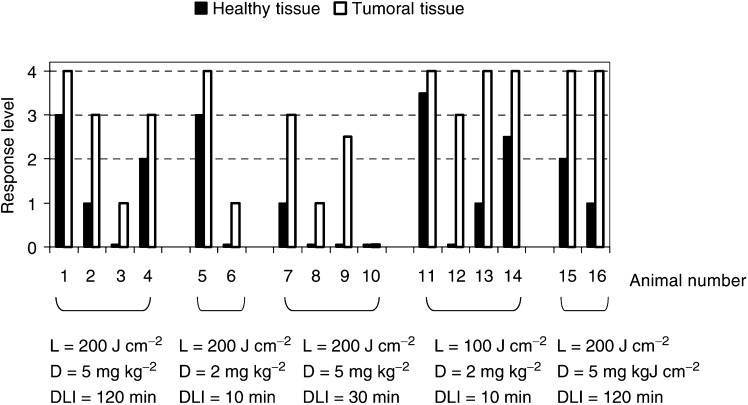
Response level of the tumour and contralateral healthy tissue after PDT.

The induced squamous cell carcinoma were treated when the tumours began to be invasive and exophytic, with a size ranging between 2 and 8 mm in diameter. The contralateral irradiation of the cheek pouch allows a direct comparison of the PDT treatment between healthy tissue and tumoral tissue. In order to study the selectivity of the effect of Tookad® on tumoral and normal tissue, the PDT treatment was conducted simultaneously on both cheek pouches of the hamster, that is, at the same DLI. The contralateral irradiation allows a direct comparison of the level of damage on the tumour and on the healthy tissue. The conditions of PDT treatment for the squamous cell carcinoma were chosen according to the results obtained on the normal cheek pouch in order to produce only limited damage to the normal tissue. Therefore, only light doses of 100 and 200 J cm^−2^ and drug doses of 5 and 2 mg kg^−1^ were chosen. The response levels for the PDT-treated tumour and the contralateral healthy tissue are presented in Figure 5

for all the conditions producing a detectable effect. A response level ⩾3, corresponding to the lowest tissue reaction generating a destruction of the tumour, was observed in 11 out of 16 animals for the following conditions:

*D*=5 mg kg^−1^, *L*=100 J cm^−2^, DLI=30 and 10 min,
*D*=5 mg kg^−1^, *L*=200 J cm^−2^, DLI=120 min
*D*=2 mg kg^−1^, *L*=200 J cm^−2^, DLI=30 and 10 min.

**Figure 6:**
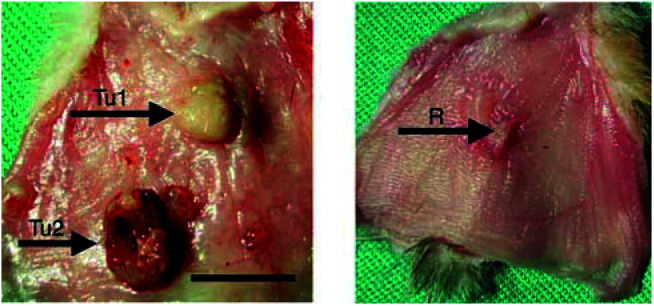
Resection of the cheek pouch 4 days after PDT at drug dose of 5 mg kg^−1^, light dose of 100 J cm^−2^, DLI=30 min. *Left*: Tul tumour irradiated with total necrosis level 4, Tu2 tumour not irradiated. *Right*: R, Reference irradiation on healthy tissue, level 2. The bar represents 10 mm.

**Figure 7:**
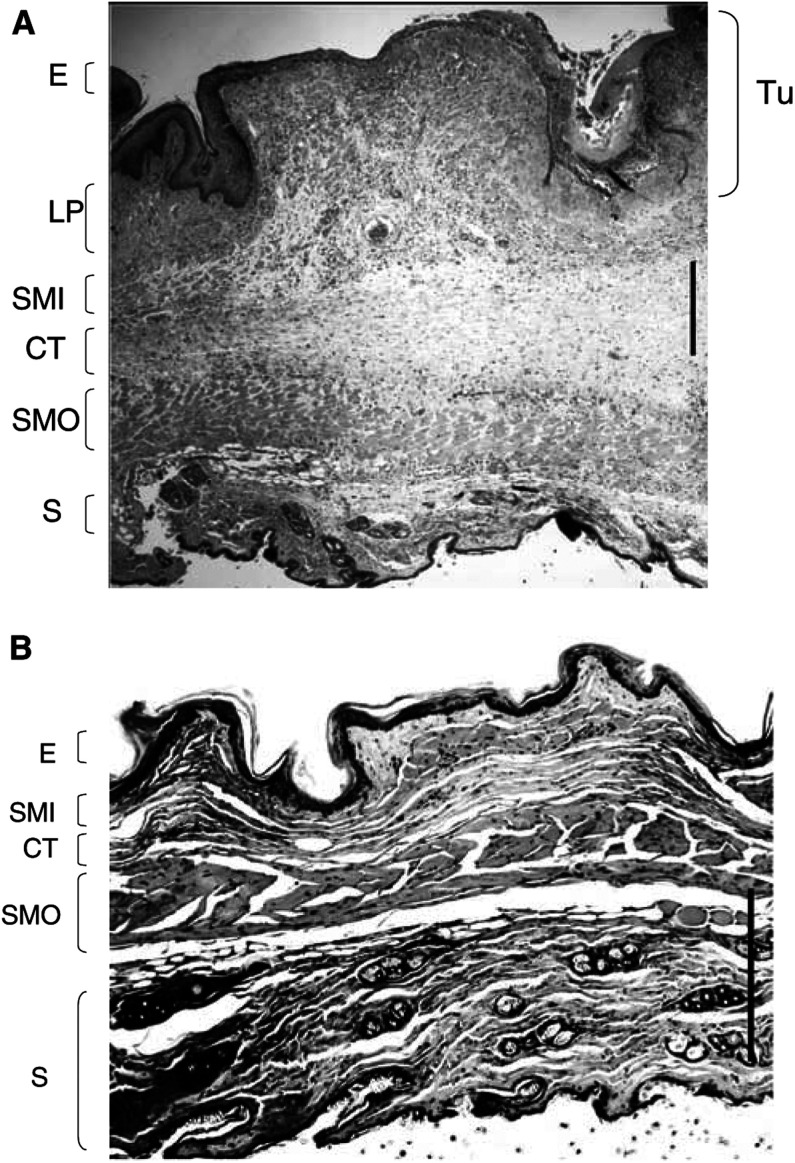
Histological section of the cheek after PDT. Light dose 100 J cm^−2^, drug dose 5 mg kg^−1^, DLI=30 min. (**A**) Tumour (Tu) with oedema, necrosis of the E, LP, striated muscle inner (SMI) and outer (SMO) layer, and CT (level 3). The tumour contains dead cells and is invaded by neutrophils. (**B**) Contralateral normal cheek pouch (level 1) The bar represents 400 *μ*m.

An example of the macroscopic response with necrosis of a tumour is given in Figure 6

(*D*=5 mg kg^−1^, *L*=100 J cm^−2^, DLI=30 min), the contralateral cheek pouch shows a response of level 2. An histological section of tumour and of its contralateral mucosae are given in Figure 7

(*D*=5 mg kg^−1^, *L*=100 J cm^−2^, DLI=30 min). The tumour contains essentially dead cells and an accumulation of neutrophils, whereas the contralateral mucosae only shows level 1 damage in the diffuse connective tissue with minimal presence of neutrophils. It is observed that the scattering of the responses was twice higher in the simultaneous contralateral irradiation experiment than in the single irradiation on the healthy tissue. The statistical analysis of the levels of response between the matched data for tumoral and normal tissue (presented in Figure 5) in all the hamsters with induced carcinoma shows a significant difference following the Wilcoxon matched-pairs signed-rank test (*α*≤0.001).

In order to determine if a lower threshold level of response could be observed in the tumoral tissue, two conditions of irradiation were chosen just below the threshold response for healthy tissue: *L*=200 J cm^−2^, *D*=2 mg kg^−1^ and DLI of 120 min, and *L*=200 J cm^−2^, *D*=1 mg kg^−1^ and DLI of 30 min. In both conditions, no significant response on either tumoral or healthy tissue was observed. This reveals that the threshold of response for the squamous cell carcinoma could not be detected at a significantly lower level than on the healthy mucosae for irradiation at 200 J cm^−2^ within the resolution of our experimental model.

## DISCUSSION

In the present preclinical study, we measured the photodynamic activity of Tookad® in terms of the three critical parameters used in clinical phototherapy: drug dose, light dose and drug–light interval. In clinical applications, it is essential to adjust these parameters to get a specific level of tumour response and to improve the therapeutic results, while minimising the risk of complications. The hamster cheek pouch allows controlled dosimetry on a superposition of histologically different layers of tissue, which permits the study of the level of necrosis following PDT. The steep decay of the PDT response, in terms of the injection–irradiation interval, is dependent on the drug dose and the light dose. This on/off character in this animal model makes the drug dose, the light dose and the DLI critical parameters. The very fast decay of the response and the absence of response for DLI of 240 min at a light dose of 300 J cm^−2^ and a drug dose of 5 mg kg^−1^ are characteristic of a predominantly vascular effect. This fast decay is in contrast to the methyl ester of bacteriopheophorbide a, which has a concentration in the tumour peaking at 8 h after injection (Ismail *et al*, 1998).

A contrast of the damage toward vascularised connective tissue and striated muscle was observed, the Tookad® inducing first an effect at the lowest drug–light doses in the diffuse connective tissue and in the striated muscle before acting on the epithelial tissue as shown by the cell death beginning on the inside of the mucosae. The vascularised diffuse connective tissue is essential to nourish and maintain the tissular integrity of the whole mucosae. Therefore, at a higher level of damage, this observation suggests that the epithelia are necrosed essentially due to the early necrosis of the submucosal layers. This result is in contrast to other PSs such as mTHPC, which provide a contrast in favour of the epithelial tissue at long DLI (>50 h). It should be noted that this 50-h DLI is much longer than those considered in the present study (Andrejevic-Blant *et al*, 1997a,^2000^).

The rapid elimination of Tookad® is an advantage for several clinical applications and reduces the side effects. Rapid blood clearance was already observed for other PSs such as benzoporphyrin derivative (BPD-MA). Therefore, Tookad® might be well-suited to the treatment of lesions sensitive to the neoangiogenesis or neovascularised tumour as well as already vascularised tumour (Los and Voest, 2001). The treatment of atherosclerotic plaques (Yamaguchi *et al*, 2001) with other vascular PSs has shown promising results on animal models, as well as on chronic inflammatory reaction in rheumatoid arthritis (Chowdhary *et al*, 1998; Trauner *et al*, 1998; Hendrich *et al*, 2000). These pathologies are potential targets for PSs with a fast clearance such as Tookad®. Tookad® can be activated at relatively longer wavelengths than other PS, which permits deeper light penetration into the tissues. In addition, this opens the possibility of Tookad® being useful in treating pigmented tissues, which have relatively smaller absorptions at longer wavelengths (Zilberstein *et al*, 2001).

The hamster cheek pouch is a good model for squamous cell carcinoma and mimics well the neoplasic development of the human aerodigestive tract (Andrejevic *et al*, 1996). The matched pair comparison of the tumoral *vs* healthy mucosae shows a significantly higher level of damage to the tumour. Therefore, a higher response of the necrotic damage toward the tumour *vs* the normal tissue can be obtained. These results can be attributed to the vascularisation of the induced tumour, which makes the tumour particularly sensitive to destruction by the PS present in the blood compartment However, no significant difference in the threshold of response (level of damage ⩾1) for the drug dose and the light dose could be highlighted in the tumoral *vs* healthy tissue. A tissular or a cellular accumulation of the PS in the neoplasic tissue should reveal a lower threshold of response compared to the normal tissue, ideally allowing the treatment of tumours without affecting the surrounding healthy tissue. In order to lower the threshold of response, an improved targeting of tumour tissue is desirable. This may be achieved by coupling Tookad® to molecules, which may achieve better selectivity in neoplasic tissue (Folli *et al*, 1992).

Some critical remarks about the extension of the results obtained in such animal models have to be kept in mind for future clinical applications. The binding of the PS molecules to plasma protein after injection can be different in human and in the hamster. This is of importance as the distribution of the PS in the different physiological, tissular and subcellular compartments (MacDonald *et al*, 1999) is strongly dependent on the degree of molecular microaggregation and on the injected formulation of the PS.

In conclusion, Tookad® presents the profile of a PS with predominantly vascular effects under the conditions of our experiment, with a fast decay of the PDT response in terms of the drug–light interval. A higher level of response was observed towards vascularized tumours *vs* normal tissue. Neovascularisation of tissue is essential for the growth of a solid tumour. The concept of a near-infrared absorbing PS with predominantly vascular localisation is an interesting potential treatment strategy.

## References

[bib1] Andrejevic S, Savary JF, Fontolliet C, Monnier P, van den Bergh H (1996) 7,12-Dimethylbenz[a]anthracene-induced ‘early’ squamous cell carcinoma in the Golden Syrian hamster: evaluation of an animal model and comparison with ‘early’ forms of human squamous cell carcinoma in the upper aero-digestive tract. Int J Exp Pathol 77(1): 7–14 8664146 10.1046/j.1365-2613.1996.956095.x PMC2691614

[bib8] Andrejevic-Blant S, Woodtli A, Wagnières G, Fontolliet C, van den Bergh H, Monnier P (1996) *In vivo* fluence rate effect in photodynamic therapy of early cancers with tetra(*m*-hydroxyphenyl)chlorin. Photochem Photobiol 64(6): 963–968 8972639 10.1111/j.1751-1097.1996.tb01862.x

[bib2] Andrejevic-Blant S, Hadjur C, Ballini JP, Wagnières G, Fontolliet C, van den Bergh H, Monnier P (1997a) Photodynamic therapy of early squamous cell carcinoma with tetra(*m*-hydroxyphenyl)chlorin: optimal drug – light interval. Br J Cancer 76(8): 1021–1028 9376261 10.1038/bjc.1997.502 PMC2228103

[bib3] Andrejevic-Blant S, Theumann J-F, Forrer M, Wagnières G, van den Bergh H, Monnier P (1997b) Wavelength-dependent effect of tetra(*m*-hydroxyphenyl)chlorin for photodynamic therapy in an ‘early’ squamous cell carcinoma model. Lasers Med Sci 12: 269–273 20803335 10.1007/BF02765108

[bib4] Andrejevic-Blant S, Woodtli A, Wagnières G, Fontolliet C, van den Bergh H, Monnier P (1998) Interstitial photodynamic therapy with tetra(*m*-hydroxyphenyl)chlorin: tumor versus striated muscle damage. Int J Radial Oncol Biol Phys 42(2): 403–412 10.1016/s0360-3016(98)00221-1 9788423

[bib5] Andrejevic-Blant S, Ballini JP, van den Bergh H, Fontolliet C, Wagnières G Monnier P (2000) Time-dependent biodistribution of tetra(*m*-hydroxyphenyl)chlorin and benzoporphyrin derivative monoacid ring A in the hamster model: comparative fluorescence microscopy study. Photochem Photobiol. 71(3): 333–340 10732452 10.1562/0031-8655(2000)071<0333:TDBOTM>2.0.CO;2

[bib6] Andrejevic-Blant S, Grosjean P, Ballini JP, Wagnières G, van den Bergh H, Fontolliet C, Monnier P (2001) Localization of tetra(*m*-hydroxyphenyl)chlorin (Foscan) in human healthy tissues and squamous cell carcinomas of the upper aero-digestive tract the esophagus and the bronchi: a fluorescence microscopy study. J Photochem Photobiol B 61(1-2): 1–9 11485842 10.1016/s1011-1344(01)00148-8

[bib7] Aprahamian M, Evrard S, Keller P, Tsuji M, Balboni G, Damge C, Marescaux J (1993) Distribution of pheophorbide A in normal tissues and in an experimental pancreatic cancer in rats. Anticancer Drug Des 8(2): 101–114 8494601

[bib9] Brown SB, Mellish KJ (2001) Verteporfin: a milestone in ophthalmology and photodynamic therapy. Expert Opin Pharmacother 2(2): 351–361 11336591 10.1517/14656566.2.2.351

[bib10] Chen Q, Huang Z, Luck D, Beckers J, Brun P-H, Wilson BC, Scherz A, Salomon Y, Hetzel FW (2002) Preclinical studies in normal canine prostate of a novel palladium-bacteriopheophorbide (WST09) photosensitizer for photodynamic therapy of prostate cancer. Photochem Photobiol 76(4): 438–445 12405153 10.1562/0031-8655(2002)076<0438:PSINCP>2.0.CO;2

[bib11] Chowdhary RK, Ratkay LG, Canaan AJ, Waterfield JD, Richter AM, Levy JG (1998) Uptake of verteporfin by articular tissues following systemic and intra-articular administration. Biopharm Drug Dispos 19(6): 395–400 9737820 10.1002/(sici)1099-081x(199809)19:6<395::aid-bdd117>3.0.co;2-9

[bib12] Folli S, Wagnières G, Pèlegrin A, Calmes J-M, Braichotte D, Buchegger F, Chalandon Y, Givel J-C, Hardman N, Heusser Ch, Chapuis G, Châtelain A, van den Bergh H, Mach J-P (1992) Immunophotodiagnosis of colon carcinomas in patients injected with fluoresceinated chimeric antibodies against carcinoembryonic antigen. Proc Natl Acad Sci USA 89: 7973–7977 1518823 10.1073/pnas.89.17.7973 PMC49837

[bib13] Fritsch C, Goerz G, Ruzicka T (1998) Photodynamic therapy in dermatology. Arch Dermatol. 134(2): 207–214 9487213 10.1001/archderm.134.2.207

[bib14] Grosjean P, Savary JF, Mizeret J, Wagnières G, Woodtli A, Theumann JF, Fontolliet C, van den Bergh H, Monnier P (1996) Photodynamic therapy for cancer of the upper aerodigestive tract using tetra(*m*-hydroxyphenyl)chlorin. J Clin Laser Med Surg 14(5): 281–287 9612194 10.1089/clm.1996.14.281

[bib15] Guillemin F, Cosserat-Gerardin I, Notter D, Vigneron C (2001) Diagnosis and treatment of bladder tumors by photodynamic therapy. Pathol Biol (Paris) 49(10): 815–823 11776693 10.1016/s0369-8114(01)00223-1

[bib16] Hajri A, Coffy S, Vallat F, Evrard S, Marescaux J, Aprahamian M (1999) Human pancreatic carcinoma cells are sensitive to photodynamic therapy *in vitro* and *in vivo*. Br J Surg 86(7): 899–906 10417562 10.1046/j.1365-2168.1999.01132.x

[bib17] Hendrich C, Huttmann G, Vispo-Seara JL, Houserek S, Siebert WE (2000) Experimental photodynamic laser therapy for rheumatoid arthritis with a second generation photosensitizer. Knee Surg Sports Traumatol Arthroscler 8(3): 90–94 10.1007/s001670050213 10883433

[bib18] Ismail MS, Dressler C, Roder B, Weitzel H, Berlien HP (1998) 13(2)-hydroxy-bacteriopheophorbide a-methylester pharmacokinetics in mice bearing Lewis lung carcinoma. Lasers Med Sci 13(1): 78–81

[bib19] Jichlinski P, Leisinger H-J (2001) Photodynamic therapy in superficial bladder cancer: past, present and future. Urol Res 29: 396–405 11828993 10.1007/s002400100215

[bib20] Karrer S, Szeimies RM, Hohenleutner U, Landthaler M (2001) Role of lasers and photodynamic therapy in the treatment of cutaneous malignancy. Am J Clin Dermatol 2(4): 229–237 11705250 10.2165/00128071-200102040-00004

[bib21] Kingsbury JS, Cecere W, Mang TS, Liebow C (1997) Photodynamic therapy for premalignant lesions in DMBA-treated hamsters: a preliminary study. J Oral Maxillofac Surg 55(4): 376–381 9120701 10.1016/s0278-2391(97)90130-0

[bib22] Los M, Voest EE (2001) The potential role of antivascular therapy in the adjuvant and neoadjuvant treatment of cancer. Semin Oncol 28(1): 93–105 11254869 10.1016/s0093-7754(01)90047-8

[bib23] MacDonald IJ, Morgan J, Bellnier DA, Paszkiewicz GM, Whitaker JE, Litchfield DJ, Dougherty TJ (1999) Subcellular localization patterns and their relationship to photodynamic activity of pyropheophorbide-a derivatives. Photochem Photobiol 70(5): 789–797 10568171

[bib24] Metz JM, Friedberg JS (2001) Endobronchial photodynamic therapy for the treatment of lung cancer. Chest Surg Clin North Am 11(4): 829–839 11780298

[bib25] Radu A, Wagnières G, van den Bergh H, Monnier P (2000) Photodynamic therapy of early squamous cell cancers of the esophagus. Gastrointest Endosc Clin North Am 10(3): 439–460 10900053

[bib26] Ritz JP, Roggan A, Isbert C, Muller G, Buhr HJ, Germer CT (2001) Optical properties of native and coagulated porcine liver tissue between 400 and 2400 nm. Lasers Surg Med 29(3): 205–212 11573221 10.1002/lsm.1134

[bib27] Rosenbach-Belkin V, Chen L, Fiedor L, Salomon Y, Scherz A (1998) Chlorophyll and bacteriochlorophyll derivatives as photodynamic agents. In Photodynamic Tumor Therapy, Moser JG (ed) pp 117–125. Amsterdam: Harwood

[bib28] Rovers JP, de Jode ML, Grahn MF (2000) Significantly increased lesion size by using the near-infrared photosensitizer 5,10,15,20-tetrakis (*m*-hydroxyphenyl)bacteriochlorin in interstitial photodynamic therapy of normal rat liver tissue. Lasers Surg Med 27(3): 235–240 11013385 10.1002/1096-9101(2000)27:3<235::aid-lsm5>3.0.co;2-t

[bib29] Savary JF, Monnier P, Fontolliet C, Mizeret J, Wagnières G, Braichotte D, van den Bergh H (1997) Photodynamic therapy for early squamous cell carcinomas of the esophagus, bronchi, and mouth with *m*-tetra (hydroxyphenyl) chlorin. Arch Otolaryngol Head Neck Surg 123(2): 162–168 9046283 10.1001/archotol.1997.01900020042006

[bib30] Scherz A, Salomon Y, Brandis A, Scheer H (2000) Palladium-substituted bacteriochlorophyll derivatives and use thereof. Publication. Steba Biotech Investigation protocols. Israel: Yeda Research and Development Co. Ltd PCT Int. Appl. 59 pp.

[bib31] Schreiber S, Gross S, Brandis A, Harmelin A, Rosenbach-Belkin V, Scherz A, Salomon Y (2002) Local photodynamic therapy of rat C6 glioma xenografts with Pd-bacteriopheophorbide leads to decreased metastases and increase of animal cure compared to surgery. Int J Cancer 99(2): 279–285 11979445 10.1002/ijc.10299

[bib32] Trauner KB, Gandour-Edwards R, Bamberg M, Shortkroff S, Sledge C, Hasan T (1998) Photodynamic synovectomy using benzoporphyrin derivative in an antigen-induced arthritis model for rheumatoid arthritis. Photochem Photobiol 67(1): 133–139 9477771

[bib33] Webber J, Herman M, Kessel D, Fromm D (1999) Current concepts in gastrointestinal photodynamic therapy. Ann Surg 230(1): 12–23 10400031 10.1097/00000658-199907000-00003 PMC1420839

[bib34] Workman P, Twentyman P, Balkwill F, Balmain A, Chaplin D, Double J, Embleton J, Newell D, Raymond R, Stables J, Stephens T, Wallace J (1998) United Kingdom Co-ordinating Committee on Cancer Research (UKCCCR) Guidelines for the Welfare of Animals in Experimental Neoplasia (Second Edition). Br J Cancer 77: 1–10 10.1038/bjc.1998.1 PMC2151254 9459138

[bib35] Yamaguchi A, Woodburn KW, Hayase M, Hoyt G, Robbins RC (2001) Photodynamic therapy with motexafin lutetium (Lu-Tex) reduces experimental graft coronary artery disease. Transplantation 71(11): 1526–1532 11435960 10.1097/00007890-200106150-00008

[bib36] Zellweger M, Radu A, Monnier P, van den Bergh H, Wagnières G (2000) Fluorescence pharmacokinetics of Lutetium Texaphyrin (PCI-0123, Lu-Tex) in the skin and in healthy and tumoral hamster cheek-pouch mucosa. J Photochem Photobiol B 55(1): 56–62 10877068 10.1016/s1011-1344(00)00027-0

[bib37] Zilberstein J, Schreiber S, Bloemers MC, Bendel P, Neeman M, Schechtman E, Kohen F, Scherz A, Salomon Y (2001) Antivascular treatment of solid melanoma tumors with bacteriochlorophyll-serine-based photodynamic therapy. Photochem Photobiol. 73(3): 257–266 11281022 10.1562/0031-8655(2001)073<0257:atosmt>2.0.co;2

